# THREE-YEAR OUTCOMES IN A RANDOMIZED SINGLE-BLIND CONTROLLED TRIAL OF INTRAVITREAL RANIBIZUMAB AND ORAL SUPPLEMENTATION WITH DOCOSAHEXAENOIC ACID AND ANTIOXIDANTS FOR DIABETIC MACULAR EDEMA

**DOI:** 10.1097/IAE.0000000000002114

**Published:** 2018-02-22

**Authors:** María Lafuente, Lourdes Ortín, María Argente, José L. Guindo, María D. López-Bernal, Francisco J. López-Román, Joan Carles Domingo, Jerónimo Lajara

**Affiliations:** * Department of Ophthalmology, Hospital Universitario Morales Meseguer, Murcia, Spain;; † Faculty of Health Sciences, San Antonio Catholic University of Murcia, Murcia, Spain; and; ‡ Department of Biochemistry and Molecular Biology, Faculty of Biology, University of Barcelona, Barcelona, Spain.

**Keywords:** diabetic macular edema, docosahexaenoic acid, essential fatty acids, intravitreal ranibizumab, omega-3 polyunsaturated fatty acids, nutraceutics

## Abstract

**Purpose:**

To report 3-year results of a randomized single-blind controlled trial of intravitreal ranibizumab combined with oral docosahexaenoic acid (DHA) supplementation versus ranibizumab alone in patients with diabetic macular edema.

**Methods:**

There were 26 patients (31 eyes) in the DHA group and 29 (38 eyes) in the control group. Ranibizumab (0.5 mg) was administered monthly for the first 4 months followed by a pro re nata (PRN) regimen. In the experimental group, patients received oral DHA supplementation (1,050 mg/day) (Brudyretina 1.5 g).

**Results:**

At 36 months, mean decrease of central subfield macular thickness was higher in the DHA-supplementation group than in controls (275 ± 50 *μ*m vs. 310 ± 97 *μ*m) with significant differences at Months 25, 30, 33, and 34. Between-group differences in best-corrected visual acuity were not found, but the percentages of ETRDS gains >5 and >10 letters were higher in the DHA-supplementation group. Differences serum HbA1c, plasma total antioxidant capacity values, erythrocyte DHA content, and serum IL-6 levels were all significant in favor of the DHA-supplementation group.

**Conclusion:**

The addition of a high-rich DHA dietary supplement to intravitreal ranibizumab was effective to achieve better sustained improvement of central subfield macular thickness outcomes after 3 years of follow-up as compared with intravitreal ranibizumab alone.

## Abstract 2

The decrease in macular thickness observed at 24 months was maintained at Month 36 in patients with diabetic macular edema treated with a combination of intravitreal ranibizumab and oral supplementation with high-dose docosahexaenoic acid and antioxidants.

Diabetic macular edema (DME), a severe form of diabetic retinopathy (DR), is the leading cause of vision loss in working age adults in developed countries.^1^ The disease has multifactorial and interconnected pathophysiological mechanisms with poor glycemic control considered of major importance. Hyperglycemia, endothelial dysfunction, retinal hypoxia and oxidative stress, local inflammatory activity with upregulation of cytokines and growth factors, microaneurysms, breakdown of the blood–retinal barrier, and retinal neurodegeneration have been shown to be involved in the development and progression of DR.^2,3^ However, the molecular basis of multiple cellular mechanisms affecting the neurovascular unit remains to be elucidated.

Although advances in understanding the etiopathogenesis of DME have already translated into new effective therapeutic interventions, such as intravitreal treatment with antiangiogenic drugs, further options for optimizing outcomes are continually pursued. Dietary supplementation with omega-3 long-chain polyunsaturated fatty acids (ω-3 PUFAs) has emerged as one of these alternatives based on the significant role of docosahexaenoic acid (DHA), the dominant fatty acid of retinal phospholipids, in maintaining retinal integrity.^4–6^ The pleiotropic effects of DHA including antiinflammatory, antioxidant, antiproliferative, and antiangiogenic properties may play an important role in modulating the production of proinflammatory cytokines and proangiogenic factors, the effect of oxidative stress damage of retinal pigment epithelial cells, and the intrinsic mechanisms of other contributing pathways to endothelial vascular dysfunction in the diabetic retina.^7–11^

The effectiveness of intravitreal ranibizumab combined with a dietary supplement rich in DHA plus antioxidants in patients with DME was previously evaluated in a 2-year randomized single-blind controlled trial.^12^ Combined treatment was significantly superior to ranibizumab alone in reducing central macular thickness with a trend of amelioration of visual acuity. We report the 3-year results of this study.

## Patients and Methods

### Design and Patients

This randomized single-blind controlled trial was approved by the Ethics Committee of Hospital Universitario Morales Meseguer of Murcia, Spain, and was registered at https://eudract.ema.europa.eu/ (EudraCT trial number 2015-001082-74). All participants gave written informed consent.

Patients' eligibility has been previously described.^12^ Briefly, adult patients with type 2 diabetes who presented with decreased vision due to central-involved DME (defined as retinal thickening involving the 1-mm central optical coherence tomography subfield thickness) were eligible. All patients received four loading doses of ranibizumab (0.5 mg/0.05 mL, Lucentis; Novartis Farmacéutica, Barcelona, Spain) during the first 4 months and then treated on as-needed (pro re nata [PRN]) basis. The improvement was defined as a gain of 5 or more ETDRS letters of best-corrected visual acuity (BCVA) and/or a 100-*μ*m decrease in central subfield macular thickness (CSMT) measurement by optical coherence tomography as compared to the previous visit. There was no change in stability over the last 3 monthly consecutive visits. Criteria for retreatment were loss of stability in terms of a difference in BCVA ≤5 ETDRS letters and a difference in CSMT ≥100 *μ*m. All candidates for retreatment received two loading doses of ranibizumab and were further evaluated.

Patients were randomized using a table of random numbers to intravitreal ranibizumab either with oral DHA supplementation (intervention group) or without oral DHA supplementation (control group). All patients received four loading doses of ranibizumab (0.5 mg/0.05 mL, Lucentis; Novartis Farmacéutica, Barcelona, Spain) during the first 4 months and then were treated on as-needed (pro-re-nata) basis. Patients in the intervention group received a high-rich DHA (1,050 mg/day) nutraceutical formulation (Brudyretina 1.5 g; Brudy Lab, S.L, Barcelona, Spain). This is a concentrated DHA triglyceride having a high antioxidant activity patented to prevent cellular oxidative damage.^13,14^ It includes a high dose of DHA (1 g), eicosapentaenoic acid, a mixture of B vitamins, vitamins C and E, lutein, zeaxanthin, and minerals. Patients were instructed to take 3 capsules of Brudyretina 1.5 g once daily. The treatment evaluator (M.L.) was masked about which subjects were receiving DHA supplementation. The safety evaluator (J.L.G.) was not masked about treatment assignment but was unaware of results of outcome variables.

### Outcome and Study Procedures

Outcome variables included the number of ranibizumab intravitreal injections; BCVA (ETDRS optotype at 2 m distance from the observer); CSMT (Stratus OCT; Carl Zeiss Meditec, Dublin, CA); serum levels of glycosylated hemoglobin (HbA1c); plasma total antioxidant capacity (TAC) (OxiSelect Total Capacity Assay kit, STA-360; Cell Biolabs Inc, San Diego, CA); plasma levels of interleukin 6 (IL-6) (Human IL-6 ELISA kit; Cat. No 950.030 purchased from Diaclone SAS, Besancon Cedex, France); and erythrocyte membrane content of ω-3 DHA using the method of Lepage and Roy.^15^ All variables were measured at the baseline and at 12, 24, and 36 months, except for IL-6, which was measured at the baseline and at 36 months. Details of laboratory techniques have been previously reported.^12^

Patients were visited at the outpatient ophthalmology clinic every month. At each visit, BCVA and measurement of CSMT was performed. Also, at each visit, the nutraceutical supplement was delivered to the patient for 1-month treatment. Compliance with DHA supplementation was assessed at the study visits and by a telephone call approximately at 15-day intervals.

### Statistical Analysis

A sample of 44 patients per treatment group was required (total 88 patients), according to an expected mean difference of 75 *μ*m in CSMT between groups, assuming a SD of 135 *μ*m^16^ and a dropout rate of 10%. The eye was the unit of analysis for CSMT, BCVA, and ETDRS change of BCVA. When both eyes were affected, they were included in the same study group. Categorical data are expressed as frequencies and percentages and continuous data as mean and ± SD and 95% confidence interval (CI). The chi-square (χ^2^) test or the Fisher exact tests were used for the comparison of categorical variables between the study groups. Mixed linear model analysis was used to assess differences in BCVA, CSMT, and HbA1c levels between the study groups throughout the 34-month study period (covariate: baseline BCVA, CSMT, or HbA1c; random factor: patients), using the mean between the anterior and posterior measured values for missing data. If no posterior measured values were available, the anterior value was used for analysis. All analyses in the 36-month extension study are presented by treatment groups as in the 2-year core trial, namely, ranibizumab + DHA supplementation and ranibizumab alone. Data are summarized for 12-, 24-, and 36-month time periods. Changes of BCVA and CSMT are also presented on a monthly basis, that is, Month 1 to 36. Statistical significance was set at *P* < 0.05. Statistical analyses were performed with the Statistical Package for the Social Sciences, version 11.0 software (SPSS Inc, Chicago, IL).

## Results

**Fig. 1.**
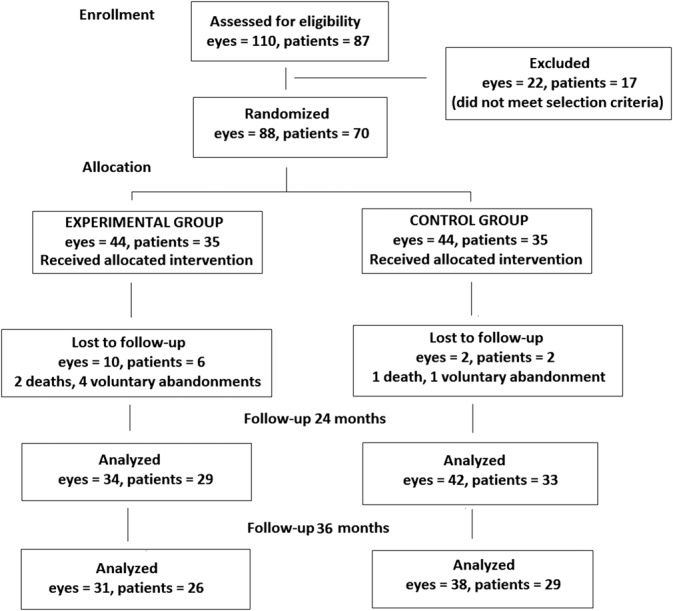
Flowchart of the study patients and eyes.

At 24 months, 76 eyes were analyzed (DHA-supplementation group 34 eyes, 29 patients; control group 42 eyes, 33 patients). A total of 15 patients were lost to follow-up from the baseline to the end of the study (DHA group, n = 9; control group, n = 6). There were 8 patients lost to follow-up from the baseline to 24 months and 7 patients from 24 to 36 months (Figure 1). Between the baseline and 24 months, 2 unrelated deaths occurred in the DHA group and in 1 in the control group. The remaining cases were voluntary abandonments. There were no significant differences in BCVA and CMST at the baseline and throughout the study between patients who abandoned the study and those who completed the 36-month follow-up period both in the DHA and control groups (data not shown). The flowchart of patients during enrollment, randomization, follow-up, and analysis is shown in Figure 1.

A total of 69 eyes (DHA-supplementation group 31 eyes, 26 patients; control group 38 eyes, 29 patients) were finally included and followed over 36 months. There were 35 men and 25 women (DHA group 17 men and 9 women; control group 18 men and 11 women) with a mean age of 67.2 ± 7.6 years (range 52–82).

**Fig. 2.**
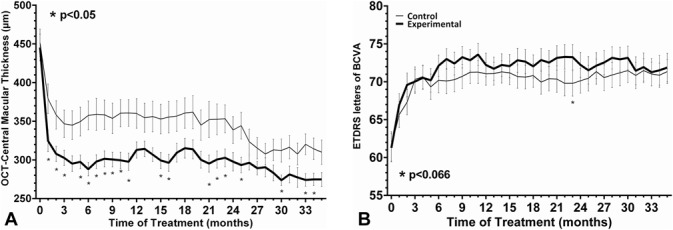
**A.** Changes of CSMT from the baseline to 36 months after treatment with DHA supplementation as compared with controls (asterisk indicates statistically significant differences *P* < 0.05) (DHA-supplementation group: black line; control group: gray line). **B.** Changes in ETDRS BCVA letters in the DHA-supplementation group and in controls (DHA-supplementation group: black line; control group: gray line).

In the DHA-supplementation group, the mean CSMT at the baseline of 444 ± 98 *μ*m decreased to 301 ± 67 *μ*m at 24 months and 275 ± 50 *μ*m at 36 months. In controls, the mean CSMT at the baseline was 450 ± 112 *μ*m and 345 ± 108 *μ*m at 24 months and 310 ± 97 *μ*m at 36 months. In both groups as compared with baseline, the ∆ mean changes of CSMT at 12, 24, and 36 months were statistically significant (*P* < 0.001) for within group comparisons, whereas between-group comparison were only significant (*P* = 0.024) at 24 months (Table 1). As shown in Figure 2A, differences in CSMT between the DHA-supplementation group and the control group were statistically significant at months 25 (*P* = 0.024), 30 (*P* = 0.05), 33 (*P* = 0.011), and 34 (*P* = 0.039).

**Table 1.**
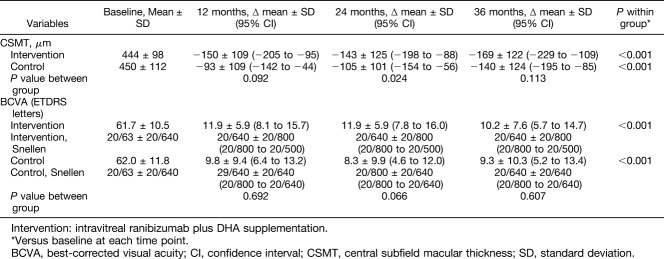
Changes of CSMT and BCVA at 12, 24, and 36 Months in the DHA-Supplementation Control Groups

Changes in BCVA between 24 and 36 months in the DHA-supplementation group and in the control group were not observed (Figure 2B). The mean BCVA measure at the baseline in ETDRS letters was 61.7 ± 10.5 (20/63 Snellen) in the DHA-supplementation group and 62.0 ± 11.8 (20/63 Snellen) in the control group (*P* = 0.607). Also, in both groups and as compared with baseline, the ∆ mean changes of BVCA at 12, 24, and 36 months were statistically significant (*P* < 0.001) but were not significant between-group comparisons (Table 1).

Differences in changes of ETDRS letters during the study period are shown in Table 2. Although statistically significant differences between the study groups were not found, the percentages of ETRDS gains >5 and >10 letters at 36 months were higher in the DHA-supplementation group than in controls (77.4% vs. 73.7% [3.7% difference] and 61.3% vs. 44.8% [16.5% difference], respectively). ETDRS gains of >15 letters were recorded in 21.1% of patients in the control group and in 19.4% of patients in the DHA-supplementation group (1.7% difference). However, none of the patients in the DHA-supplementation group lost >5 ETRDS letters, and the percentage of eyes with stable BCVA (ETDRS change −5, +5 letters) was higher in the DHA group.

**Table 2.**
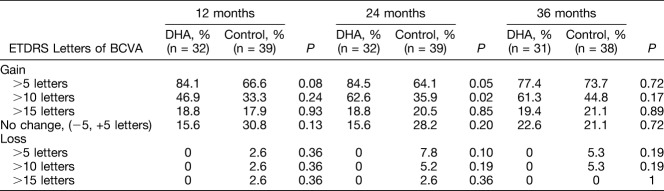
Changes of ETDRS Letters Throughout the 3-Year Study Period

**Fig. 3.**
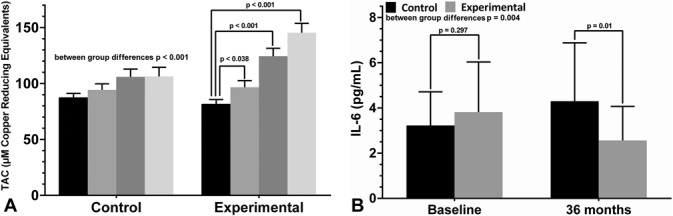
**A.** Increases in TAC in the two study groups (basal: black bar; 12 months: gray bar; 24 months: dark gray bar; 36 months: light gray bar). **B.** Changes of serum IL-6 levels in the experimental (DHA supplementation) and control groups (basal: black bar; 36 months: gray bar).

Results of HbA1c, TAC, DHA erythrocyte content, and IL-6 are shown in Table 3. At 36 months, patients in the DHA-supplementation group showed a better metabolic control with significantly lower serum levels of HbA1c than controls (7.13 ± 0.94% vs. 7.79 ± 1.48%, *P* = 0.05) (between-group differences *P* = 0.035). TAC levels increased in both study groups, but the magnitude of increases was higher in the DHA-supplementation group (between-group differences *P* = 0.001). Significant differences observed in the DHA group at 12 and 24 months as compared with baseline were maintained at 36 months (Figure 3A). In relation to serum IL-6 levels, mean values decreased from 3.81 ± 2.22 pg/mL at the baseline to 2.56 ± 1.51 at 36 months, whereas increased from 3.22 ± 1.49 pg/mL to 4.30 ± 2.58 pg/mL in the control group (between-group differences *P* = 0.004) (Figure 3B). The erythrocyte content of DHA only increased in the DHA-supplementation group (between-group differences *P* < 0.001).

**Table 3.**
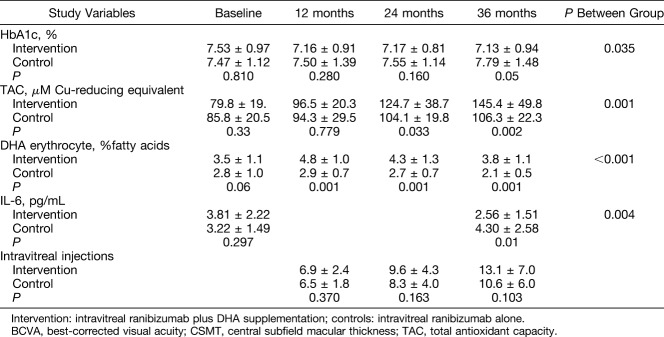
Results of Study Variables at 12, 24, and 36 Months in the DHA-Supplementation Control Groups

As shown in Table 3, there were no statistically significant differences in the number of intravitreal injections at each time interval between patients in the DHA-supplementation group and controls. The mean number of ranibizumab injections was 6.7 ± 2.2 during the first year, 2.2 ± 2.5 during the second year, and 2.8 ± 2.0 during the third year. Between 12 and 24 months, the mean ± SD (95% CI) number of injections were 2.7 ± 2.7 (1.9–3.5) in the DHA group vs. 1.8 ± 2.8 (1.2–2.4) in controls (*P* = 0.140). Between 24 and 36 months, the corresponding figures were 3.5 ± 3.3 (2.5–4.5) versus 2.3 ± 2.8 (1.7–2.9) (*P* = 0.110), respectively. A total of 50.6% of patients did not require intravitreal ranibizumab treatment from the second year onwards.

Intravitreal injections of ranibizumab were well tolerated. Between 24 and 36 months, only 2 cases of minor transient ischemic attacks were recorded in 2 patients assigned to ranibizumab treatment alone. No local adverse events were reported.

## Discussion

The 3-year results of the present randomized single-blind and controlled trial performed in routine clinical practice provide evidence of sustained anatomical improvement in patients with DME treated with intravitreal ranibizumab and oral supplementation with a combination of high-dose DHA and antioxidants. Reductions of CSMT observed over 2 years were maintained from Month 24 to 36. Interestingly, the decrease of serum IL-6 levels found at Month 36 as compared with the baseline and significantly higher than in controls is indicative of the antiinflammatory effect of DHA. These findings were associated with a sustained increase in plasma TAC levels over a period of 3 years in the DHA-supplementation group, which is consistent with the high antioxidant capacity of the nutraceutical formulation used in the present study. Increased content of DHA in the erythrocyte membrane was an indicator of the bioavailability of the oral DHA supplementation throughout the study. Also, higher levels of HbA1c suggest a better metabolic control in the DHA-supplementation group. Taken together, it seems reasonable to suggest a complementary long-term beneficial effect of oral supplementation with high-rich ω-PUFAs to intravitreal anti-VEGF therapy in patients with DME. As far as we are aware, no previous study in real-life clinical practice has assessed the effectiveness of ranibizumab over 3 years in the management of patients with DME. However, the role of long-term DHA supplementation in this setting has not been previously examined.

Certainly, significant changes in visual function did not occur, although a trend of amelioration of BCVA achieved at Months 12 and 24 was also apparent at Month 36. Although improvement of BCVA was somewhat similar in the 2 treatment groups, the proportion of patients gaining >5 and >10 ETRDS letters was greater in the DHA-supplementation group. Of note, no patient treated with ranibizumab + DHA supplementation lost >5 or >10 letters in comparison with a few patients with visual worsening in the control group. However, any inference of the potential value of DHA supplementation therapy in contributing to reducing the risk of significant ETDRS loss is inappropriate. The 36-month results from the RIDE and RISE Phase III clinical trials showed a significantly reduced incidence of further vision loss in ranibizumab-treated eyes as compared with the sham injection group.^17^ Also, in the RESTORE extension study, intravitreal ranibizumab was effective in maintaining BCVA outcomes.^18^

DHA has an inhibitory effect on the activation of NF-kB, which is responsible for the synthesis and inflammatory cytokines and vascular adhesion molecules as well as the synthesis of metalloproteinases and VEGF, a crucial proangiogenic factor driving retinal neovascularization.^19,20^ Also, the capacity of DHA in generation of eicosanoids and stimulation of proinflammation resolution docosanoids (resolvins, protectins) is consistent with a deficiency of antiinflammatory bioactive lipids, as has been suggested in the pathogenesis DME.^21^ The ability of ω-3 PUFAs, lipoxins, resolvins, and protectins to suppress IL-6, TNF-α, and VEGF production, as well as free radical generation and to restore antioxidant homeostasis in diabetic models is in line with a close association among PUFAs and their products and DR.^22–24^

Experimental evidence suggesting dietary long-chain ω-3 PUFA protection against DR has been successfully translated into clinical outcomes. In the framework of a randomized clinical trial of Mediterranean diet for primary cardiovascular prevention (PREDIMED) in subjects with Type 2 diabetes older than 55 years,^25^ researchers designed a prospective substudy to assess incident DR requiring laser photocoagulation, vitrectomy, and/or antiangiogenic treatment in 2,611 participants meeting dietary recommendation of long-chain ω-3 PUFAs of at least 500 mg/day. During a median follow-up of 6 years and after adjusting for age, sex, intervention group, and lifestyle and clinical variables, participants meeting the recommendation at the baseline compared with those not fulfilling this recommendation (<500 mg/day) showed a 48% relatively reduced risk of incident sight-threatening DR, with a hazard ratio of 0.52 (95% CI, 0.31–0.88; *P*  = 0.001).^25^ Intake of 500 mg/day of ω-3 PUFAs is easily achievable with 2 weekly servings of oily fish. A recent systematic review and meta-analysis based on 672 participants from 17 studies demonstrated that fish oil supplementation could reduce the risk of insulin resistance in subjects with metabolic syndrome by 47%,^26^ a finding that may have great implications for the prevention of Type 2 diabetes. The results of our study are one more piece of clinical data supporting the benefits of ω-3 PUFAs added to anti-VEGF therapy in patients with DME.

As previously recognized, limitations of the study include the small sample size, the single-blind design, and the lack of control over dietary intake.^12^ This was not a placebo-controlled study, and patients were not masked. In addition, it may be argued that the combination group did better because they received more ranibizumab injections, although significant differences as compared with controls were not found at any time point of the analysis. It may be speculated that the higher number of injections in the combination group might be related to more eyes fulfilling criteria for retreatment, but this possibility was not evaluated. Good adherence to DHA supplementation in the intervention group can be presumed based on the significant increase in the erythrocyte membrane content of DHA. Strengths of the study include the 3-year follow-up period and the fact that only four patients were lost between 24 and 36 months leaving a reasonable number of patients for full evaluation at 36 months.

In conclusion, in patients with DME with indication for intravitreal ranibizumab therapy, the addition of a dietary supplement rich in DHA plus antioxidant vitamins, minerals, and xanthophylls was effective to achieve better sustained improvement of CSMT outcome after 3 years of follow-up as compared with intravitreal ranibizumab alone.
